# Deep Mining of Complex Antibody Phage Pools Generated by Cell Panning Enables Discovery of Rare Antibodies Binding New Targets and Epitopes

**DOI:** 10.3389/fphar.2019.00847

**Published:** 2019-07-30

**Authors:** Anne Ljungars, Carolin Svensson, Anders Carlsson, Elin Birgersson, Ulla-Carin Tornberg, Björn Frendéus, Mats Ohlin, Mikael Mattsson

**Affiliations:** ^1^BioInvent International AB, Lund, Sweden; ^2^Department of Immunotechnology, Lund University, Lund, Sweden; ^3^Bionamic data consulting AB, Lund, Sweden

**Keywords:** therapeutic antibodies, antibody discovery, phage display, cell selection, phenotypic screening, deep mining, complex antibody phage pools

## Abstract

Phage display technology is a common approach for discovery of therapeutic antibodies. Drug candidates are typically isolated in two steps: First, a pool of antibodies is enriched through consecutive rounds of selection on a target antigen, and then individual clones are characterized in a screening procedure. When whole cells are used as targets, as in phenotypic discovery, the output phage pool typically contains thousands of antibodies, binding, in theory, hundreds of different cell surface receptors. Clonal expansion throughout the phage display enrichment process is affected by multiple factors resulting in extremely complex output phage pools where a few antibodies are highly abundant and the majority is very rare. This is a huge challenge in the screening where only a fraction of the antibodies can be tested using a conventional binding analysis, identifying mainly the most abundant clones typically binding only one or a few targets. As the expected number of antibodies and specificities in the pool is much higher, complementing methods, to reach deeper into the pool, are required, called deep mining methods. In this study, four deep mining methods were evaluated: 1) isolation of rare sub-pools of specific antibodies through selection on recombinant proteins predicted to be expressed on the target cells, 2) isolation of a sub-pool enriched for antibodies of unknown specificities through depletion of the primary phage pool on recombinant proteins corresponding to receptors known to generate many binders, 3) isolation of a sub-pool enriched for antibodies through selection on cells blocked with antibodies dominating the primary phage pool, and 4) next-generation sequencing-based analysis of isolated antibody pools followed by antibody gene synthesis and production of rare but enriched clones. We demonstrate that antibodies binding new targets and epitopes, not discovered through screening alone, can be discovered using described deep mining methods. Overall, we demonstrate the complexity of phage pools generated through selection on cells and show that a combination of conventional screening and deep mining methods are needed to fully utilize such pools. Deep mining will be important in future phenotypic antibody drug discovery efforts to increase the diversity of identified antibodies and targets.

## Introduction

Monoclonal antibodies are an important growing class of drugs (Scott et al., 2012; Ecker et al., 2015; Carter and Lazar, 2018). Such drugs can be developed through a diversity of pathways, including humanization/chimerization of monoclonal antibodies from animals and immunization of mice transgenic for human immunoglobulin gene loci. Another approach for therapeutic antibody discovery is phage display technology (Smith, 1985). Phage display has the advantage of overcoming the problem of immunological tolerance observed with immunization techniques and allows rapid discovery of antibodies against most antigens including cell surface receptors with various post-translational modifications (Bradbury et al., 2011). However, although several antibodies in the clinic, or under clinical development, derive from a phage display pipeline (Chan et al., 2014; Nixon et al., 2014; Frenzel et al., 2016), other paths for antibody discovery dominate among approved antibody drugs. One explanation for this could be that the full potential of the phage display technology remains to be exploited, and by doing so, the number of antibodies used for therapy can potentially be expanded.

Most antibody drugs have been derived through a target-based approach in which a target is selected based on a hypothesis that it has an important disease-modulating effect. Antibodies against this target are then developed. As an alternative, a phenotypic approach can be used. Here, antibodies with the desired functional effect are isolated followed by target identification. For small molecule drugs, the phenotypic approach has resulted in a large number of first-in-class drugs, where novel targets and pathways have been discovered (Swinney and Anthony, 2011; Eder et al., 2014). Implementing the phenotypic approach also for antibodies has the potential to increase target space and enable more therapeutic antibodies to be discovered. Indeed, by using this approach, antibodies against several cell types and cancer forms have been successfully isolated (Minter et al., 2017), including our discovery of antibodies against chronic lymphocytic leukemia (CLL) (Ljungars et al., 2018) and B-cell lymphoma (Fransson et al., 2006; Veitonmaki et al., 2013). Others (Rust et al., 2013; Sandercock et al., 2015; Williams et al., 2016) have used a phenotypic approach and discovered antibodies against non-small-cell lung carcinoma cells, regulatory T cells, and a breast cancer cell line (MD-MB-231). However, although successful, only a small number of targets have been found in each study, which also points in the direction that the full potential of the phage display technology, here in combination with phenotypic discovery, remains to be explored.

**Figure 1:**
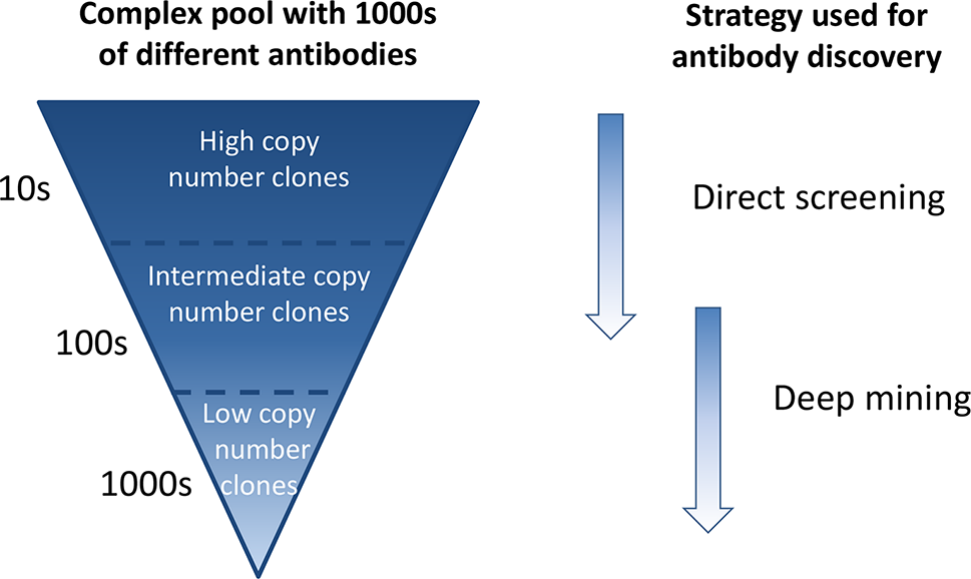
Schematic overview of a complex antibody phage pool generated through consecutive cell pannings and strategies for discovery of antibodies from such a pool. Antibodies of high frequency are easily found by direct screening of randomly selected clones, whereas low-abundant antibodies require a deep mining step prior to screening.

In phage display antibody discovery, antibodies binding to a target antigen or a target cell are enriched through several consecutive pannings with amplification of phages in between (Lee et al., 2007; Alfaleh et al., 2017). Enrichment of binders against a single target antigen is affected by several factors, including antigen concentration, antigen accessibility, antibody affinity, antibody display on phage, and phage amplification in bacteria (Bradbury and Marks, 2004; Derda et al., 2010), many of which are not directly related to antibody functionality. This results in a phage pool where some antibodies are very dominant and present at high frequencies whereas others are found at low frequencies (Ravn et al., 2010; Ravn et al., 2013). In phenotypic phage display discovery, whole cells can be used as target. We have previously shown that this approach enables discovery of antibodies against cell surface receptors present in many and few copies per cell (Ljungars et al., 2018). Therefore, when using whole cells as target, the output phage pool becomes even more complex than when using a single target antigen, as antibodies are potentially enriched against hundreds of receptors present at different levels on the cell surface. Traditional immunochemical screening, where a few thousand randomly selected antibodies from such an extremely complex output pool are tested, in this study denoted “direct screening,” will primarily identify the most frequent antibodies and specificities, since only a small subset of the antibody diversity will be covered (**Figure 1**).

To fully utilize the size and thereby the potential of phage display libraries, there is a need to develop deep mining methods that allow discovery of antibodies of low abundance. To set up such deep mining methods, we have used a phage pool that has previously been generated using our antibody library, n-CoDeR^®^, (Soderlind et al., 2000) subjected to differential cell panning on CLL cells as target, using B-cell-depleted peripheral blood mononuclear cells (PBMCs) as non-target for negative selection pressure (Ljungars et al., 2018). Screening of clones from this pool has identified antibodies targeting six cell surface receptors, CD21, CD23, CD32, CD72, CD200, and HLA-DR.

In this study, we show that there are indeed additional antibodies to be found in the anti-CLL phage pool, targeting additional receptors as well as new epitopes on antigens for which binders had already been discovered in the past. Several deep mining strategies for discovery of low-abundant clones are evaluated with respect to mining efficiency.

## Material and Methods

In this manuscript, we have used the word “antibody” to describe scFv (single-chain fragment variable) displayed on phages, soluble scFv, and IgG molecules, when it is obvious from the context which format that was used.

### Previously Generated Phage Stock

We have previously generated a phage stock through two consecutive rounds of differential cell panning using a pool of CLL cells from 10 patients as target and pooled B-cell-depleted PBMC from five healthy donors as non-target cells (Ljungars et al., 2018). This phage stock was amplified and used as input in this study.

### Overview of the Deep Mining Strategies

**Figure 2:**
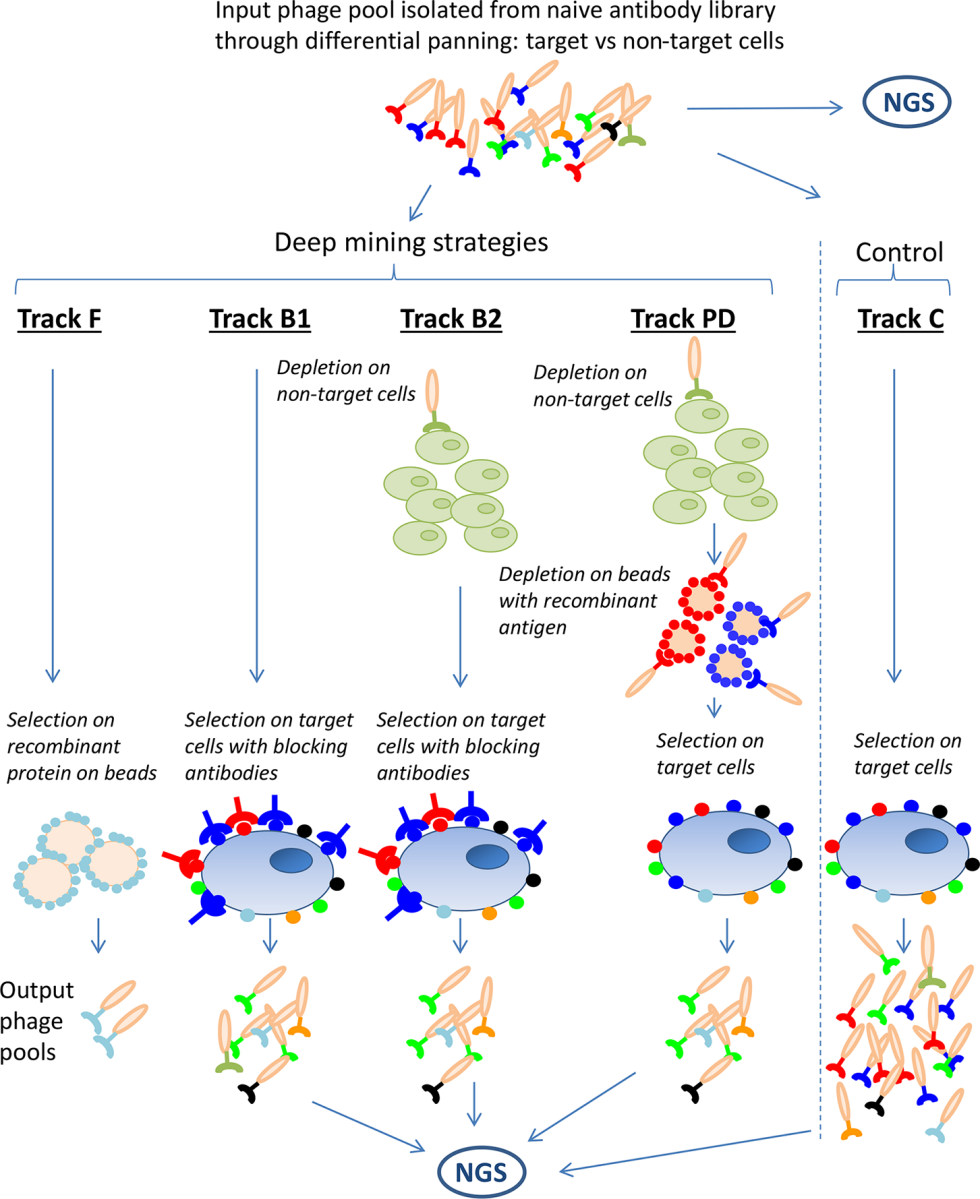
Schematic overview of the deep mining strategies used in this study. A phage pool originating from a naïve antibody library, n-CoDeR^®^, enriched for target cell binding in two consecutive selections, was used as input for various deep mining techniques including fishing (track F), antibody blocking (tracks B1 and B2), and protein depletion (track PD). A selection on target cells, without antibody blocking or protein depletion, was performed as control (track C). As an additional deep mining technique and to follow the enrichment of clone sequences in the various strategies, input and output phage pools were analyzed by next-generation sequencing (NGS).

To enable discovery of low-abundant clones, four deep mining strategies were applied on the previously generated anti-CLL phage pool. These were fishing (track F), antibody blocking (tracks B1 and B2), protein depletion (track PD), and next-generation sequencing (NGS)-based analysis followed by gene synthesis, all schematically outlined in **Figure 2**. The strategies aim to prevent the antibodies already discovered through direct screening from dominating the output pool from the deep mining selections. In track F, recombinant proteins, coated on beads were used to fish out binders from the phage pool. In tracks B1 and B2, CLL target cells were blocked with dominating antibodies from the input pool (241 IgGs discovered previously) (Ljungars et al., 2018) and then used as target in a new panning, the hypothesis being that the IgGs will block their corresponding phage from binding, favoring enrichment of new, low-abundant antibodies. In track PD, recombinant proteins corresponding to five previously discovered targets (CD21, CD23, CD32, CD72, and CD200) were used to deplete phages from the input pool prior to panning on CLL cells. A non-target cell depletion step was included in tracks B2 and PD aiming to reduce the fraction of antibodies binding to both target and non-target cells. For comparison, and as a control, a standard panning without antibody blocking or protein depletion was included (track C). In addition, the input pool and output pools from antibody blockings, protein depletion, and control were analyzed by NGS for identification of rare antibodies and to study the effect of the deep mining strategies.

### Fishing With Proteins, Track F

Five proteins with a high gene expression ratio CLL versus B-cell-depleted PBMC from healthy donors (determined by microarray analysis, Affymetrix, data not shown) were selected for fishing. Polystyrene beads (Polysciences, 17175) were coated with FCRL5, CD22 (R&D Systems, 2078-FC, 1968-SL), CD24, ROR1, or CTLA-4 (Sino Biological Inc, 11030-H02H, 13968-H08H, 11159-H08H) 25 pmol/bead, 4 beads/protein, overnight at +4°C. After washing and blocking, the input phage stock was added and incubated with the beads (1 tube/protein) overnight with rotation. After washing, bound phages were eluted with trypsin, 0.5 mg/ml (Sigma-Aldrich) for 30 min at room temperature before inactivation with aprotinin, 0.2 mg/ml (Sigma-Aldrich). The eluted phages were used to infect exponentially growing *E. coli* HB101F′ (in-house constructed from *E. coli* HB101, Thermo Fisher Scientific). The bacteria were then spread on selective agar plates and incubated overnight at +30°C before colonies were pooled and cultivated to produce a phage stock using R408 (Agilent Technologies) as helper phage.

### Depletion of the Input Phage Stock, Tracks B2 and PD

In some strategies, before panning on CLL cells, phages binding to the non-target cells or targets that generated a large number of binders in the direct screening were reduced through depletion. For a non-target cell depletion step, the input phage stock was incubated with B-cell-depleted PBMC from healthy donors for 2 h, on ice, on a rocking table. The non-target cells were then pelleted and the supernatant (with unbound phages) used for antibody blocking (track B2) or protein depletion (track PD).

For protein depletion (track PD), the extracellular domain of CD200 and CD23 (Sino Biological, 10886-H08H and 10261-H07H) were biotinylated and coupled to Streptavidin M280 Dynabeads (Thermo Fisher Scientific) (200 pmol protein to 200 µl beads) overnight. The extracellular domain of CD72 (R&D Systems, 5405-CD), CD21 (Sino Biological, 10811-H08H), and CD32 (in-house produced) were coupled to Tosylactivated M280 beads (Thermo Fisher Scientific) (200 pmol protein to 60 µl beads), according to manufacturer’s instructions and coated to an immunotube (Thermo Fisher Scientific, 470319), 70 pmol/antigen, overnight. After being washed, all beads were transferred to the coated and washed immunotube before addition of the output phages from one of the non-target cell depletions (track PD). Phages were incubated with the proteins for 2 h at room temperature before the supernatant (unbound phages) was collected and used for panning on CLL cells.

### Panning on CLL Target Cells

The preparation of CLL cells (target) and PBMC (non-target) was performed as described previously (Ljungars et al., 2018). Ethical approvals for human cells were obtained by the Ethics Committee of Skåne University Hospital. All subjects gave written informed consent in accordance with the Declaration of Helsinki. Direct screening of the input phage stock, as previously described, has resulted in discovery of antibodies binding to CD21, CD23, CD32, CD72, CD200, and HLA-DR. Two hundred forty-one of these antibodies, in human IgG1 format, were pooled together and used in the antibody blocking strategies (tracks B1 and B2). For each panning, CLL cells from seven patients were pooled and labeled with CD19 MACS beads (Miltenyi Biotec) according to the manufacturer’s instructions and incubated with the human IgG1 pool at a concentration of 10 µg of each antibody/ml (tracks B1 and B2). After 1 h incubation, the input phage stock (tracks B1 and C) or depleted stocks (tracks B2 and PD) were added and left to bind the cells for 2 h, on ice, on a rocking table. The mixtures were then loaded on MACS columns to allow extensive washing and purification of CLL cells. After elution of the CLL cells, bound phages were recovered and amplified as described above for fishing, except that 1 mg/ml of trypsin was used.

### Phage Pool Binding Analysis

Amplified phage stocks were left to bind CLL cells or B-cell-depleted PBMC for 2 h at +4°C. After washing, bound phages were detected using 10 µg/ml of Alexa Flour 647 (AF647)-labeled anti-M13 antibody (GE Healthcare, 27942001, labeled in-house) and stained with a viability dye (Fixable Viability Dye eFluor^™^ 780, eBioScience) before fixation (CellFIX, BD Biosciences) and analysis in flow cytometry (FACSVerse, BD Biosciences). The n-CoDeR^®^ library (Soderlind et al., 2000) was included as negative control.

### ScFv Binding Analysis

Genes encoding scFv were digested from the phagemid vector [purified according to the manufacturer’s instruction (Miniprep Kit, Qiagen, 27104)] and ligated into a protein expression vector (proprietary in-house). Chemically competent *E. coli* Top 10 (Thermo Fisher Scientific) were transformed and spread on selective agar plates. Single colonies were picked (Qbot, Molecular Devices) and used for production of soluble scFv carrying a 3xFLAG and a 6xHis tag in 96-well microtiter plates.

ScFv supernatants from tracks B1, B2, PD, and C were filtered (0.45 µm Millipore) and added 25 µl/well to CLL cells or B-cell-depleted PBMC (50,000 cells/well in PBS + 0.5% bovine serum albumin (BSA), 25 µl/well) and left to bind for 1 h at +4°C. Following washing, bound scFv was detected using anti-His-AF647 (R&D Systems, MAB050, labeled in-house) including a live dead marker (SYTOX green, Thermo Fisher Scientific) and analyzed in flow cytometry (HTFC, Sartorius).

ScFv from track F was analyzed for binding to recombinant proteins in enzyme-linked immunosorbent assay (ELISA). CD22, ROR1, FCRL5, and a non-target protein (carrying the same tag) were coated to ELISA plates at 0.15, 1, 2.5, or 1 pmol/well, respectively, in 50 µl/well, overnight at +4°C. After washing of plates, block buffer (PBS (Invitrogen) with 0.05% Tween 20 and 0.45% fish gelatin (both from Sigma-Aldrich), 40 µl/well, was added, and scFv supernatants, 10 µl/well, were left to bind for 1 h at room temperature. Bound scFv was then detected using an alkaline phosphatase (AP)-conjugated anti-FLAG antibody (Sigma-Aldrich) followed by addition of a luminescent substrate (CDP Star Emerald II, Thermo Fisher Scientific) and reading in a plate reader (Tecan Ultra, Tecan).

### Purification of Unique scFv

Unique scFv was identified by Sanger sequencing and re-produced, in *E. coli*, in 4-ml scale. After cultivation to exponential phase and addition of 0.5 mM IPTG (Sigma-Aldrich) to induce scFv production, production was allowed to proceed for 16 h at 25°C. Bacteria were pelleted, supernatants were discarded, and the periplasm was prepared by addition of a lysozyme-sucrose solution (1 mg/ml lysozyme (Sigma-Aldrich) in 20% sucrose (BDH Chemicals)) and 1 h incubation, with rotation at +4°C. Cell debris were removed by centrifugation, and the supernatants (containing the periplasm) were transferred to, and left to bind, a washed Ni-NTA plate (His MultiTrap HP, 96-well, GE Healthcare). After multiple washing steps, scFv was eluted using 250 mM imidazole (Sigma-Aldrich), and the concentration was measured as A_280_ (UV Star plate, Greiner), in a plate reader (Tecan Infinite F500, Tecan) before addition of protease inhibitors (NaN_3_, BDH Chemicals; and benzamidine, Sigma-Aldrich).

### Production of Human IgG1

Genes encoding the variable heavy (VH) and variable light (VL) chains of the antibody were transferred to an expression vector allowing a full-length human IgG1 to be produced as described previously (Ljungars et al., 2018).

### Dose–Response of IgG Binding in ELISA

CD22, ROR1, FCRL5, and a non-target protein with the same tag were coated at 0.2, 1, 2.5, and 1 pmol/well, respectively, to 96-well ELISA plates overnight. IgG was serially diluted in block buffer before addition to the washed plate. Bound IgG was detected with an horseradish peroxidase (HRP)-labeled anti-human F(ab)′2 antibody (Jackson ImmunoResearch, 109-036-097), followed by addition of a luminescent substrate (SuperSignal Pico 37070, Thermo Fisher Scientific). Signal was read in a Tecan Ultra plate reader.

### Blocking of Antibody Binding to Cells

Binders (human IgG1 or scFv) were serially diluted and incubated with Raji or CLL cells for 1 h at +4°C. After washing, bound antibody was detected using an allophycocyanin (APC)-conjugated anti-human IgG antibody (Jackson ImmunoResearch, 109-136-098) or anti-His-AF647 (R&D Systems, MAB050, labeled with AF647 in-house). Cells were stained for viability (SYTOX green, Thermo Fisher Scientific) and analyzed by flow cytometry (HTFC, Sartorius). For blocking, Raji or CLL cells were incubated with a polyclonal antibody (rabbit anti-CD22 (Sino Biological, 11958-T26-50), goat anti-FCRL5 (Invitrogen, PA5-48003), and goat anti-ROR1 (R&D Systems, AF2000)), 100 µg/ml, 25 µl/well, or buffer, for 1 h at +4°C. Antibodies were then added, 5 µl/well, resulting in a final concentration corresponding to the linear range of the dose–response curve and left to bind for 1 h at +4°C. After washing, bound human IgG1 or scFv was detected as described above.

### Determination of Receptor Numbers on Cells

The number of cell surface receptors for FCRL5, CD22, and ROR1 was determined using labeled antibodies (BioLegend, CD307e, 363506 and 357806) and quantification beads (Bang Laboratories, 815B) according to the manufacturer’s instruction.

### Heat Map Binding Pattern Analysis

Primary CLL cells (five patients), PBMC (two healthy donors), B-cell lines (Raji, WEHI, and RPMI8226), and other cell lines (DU145, Lovo and MCF-7) were stained with purified scFv at 10 µg/ml; and binding was detected as described above for flow cytometry. To gate the various subpopulations in PBMC, CD4-BV510, CD8-PECy7, CD19-BV510, CD14-AF488, and CD56-PE (BD Biosciences, 563094, 557746, 562440, 557700, and 555516) were added to the PBMC together with a live dead marker (eBioScience, 65-0865-14). Granulocytes were gated based on forward–side scatter profile. A hierarchical clustering of scFv based on binding pattern was done using Qlucore Omics Explorer (Qlucore, plot type heat and normalization Mean = 0, Var = 0) based on percent positive cells (compared with an isotype control).

### Target Deconvolution

All scFv was analyzed for binding to proteins corresponding to discovered specificities in ELISA. Proteins (CD72 (5405-CD), CD22 (1968-SL), FCRL5 (2078-FC), and CD45 (1430-CD-050) from R&D Systems; CD32b produced in-house; CD200 (10886-H08H), CD21 (10811-H08H), CD23 (10261-H07H), ROR1 (13968-H08H), and LY75 (16490-H08H) from Sino Biological) were coated to ELISA plates overnight. After washing of plates, scFv binding was detected as described above. Clones with a positive signal (10 times above background) in the initial test were retested in a dose–response ELISA to confirm the binding.

The specificity for some antibodies was determined through immunoprecipitation. A membrane preparation was generated using Raji cells and a plasma membrane kit according to manufacturer’s instructions (Invent Biotechnologies Minute^™^ SM-005). IGEPAL 10% (Sigma-Aldrich) in a Tris buffer (MP Biomedicals) was used for solubilization of the membrane. Antibodies, in human IgG1 format, were captured to washed protein G beads (Invitrogen, 10003D), 10 µg antibody/50 µl beads, followed by addition of the membrane preparation and incubation for 1 h at +4°C with end-over-end rotation. After washing, bound antigen and antibody were eluted from the beads by reduction (DTT, Pierce, 20291) and heating, 90°C, 5 min, before the eluted sample was loaded on a precast SDS gel and run for 5 min at 200 V. The sample was finally excised from the gel and sent to Alphalyse (Odense, Denmark), for nano-LS-MS analysis according to their standard procedure.

### Production of Individual scFv Displayed on Phages

Clones that were selected for additional testing as displayed on phage were cultured overnight followed by DNA purification (Miniprep Kit, Qiagen, 27104) according to the manufacturer’s instructions. The genes encoding scFv was digested from the expression vector (proprietary in-house) and ligated into a phagemid vector followed by transformation of chemically competent HB101F′ (in-house constructed from *E. coli* HB101, Thermo Fisher). Single transformants were used to inoculate an overnight culture. After overnight incubation, 37°C, 250 rpm, the bacteria were used to prepare individual glycerol stocks, which were used to inoculate culture medium. Cultures were grown until exponential phase when helper phage, R408, 6 × 10^9^ PFU/ml (Agilent Technologies), was added followed by 30 min incubation to allow infection to proceed before 100 µM of IPTG (Sigma-Aldrich) was added to induce protein production. After an overnight incubation, 25°C, 200 rpm, phages were concentrated through precipitation (Polyethylene glycol 6000, BDH Chemicals).

### Epitope Mapping Using scFv Displayed on Phages

ELISA plates were coated overnight with CD72 and CD21. Phages were serially diluted and added to the washed plate. Bound scFv displayed on phages were detected using an anti-M13-HRP (GE Healthcare) antibody followed by addition of a luminescent substrate (Thermo Fisher Scientific, 37070), and finally, the plates were read in a plate reader (Tecan Ultra, Tecan). A phage concentration in the linear range of the dose–response curve was used for the blocking experiment. For blocking, the pool of human IgG1, used for blocking in the panning, 10 µg/ml and IgG, or only buffer, was added to the coated and washed plates. After 1 h incubation at room temperature, phages were added into the wells containing the human IgG1 pool or buffer. After incubation and washing, bound scFv displayed on phages was detected as described above.

### Next-Generation Sequencing

Bacterial phage stocks from the input and output of all panning strategies were used to purify phagemid DNA according to manufacturer’s instructions (Miniprep Kit, Qiagen 27104) followed by PCR amplification of the scFv genes, variable heavy chain (VH) and variable light chain (VL), including complementarity-determining region heavy chain 3 (CDRH3), separately with a limited number of cycles. The PCR products were purified from an agarose gel according to manufacturer’s instructions (MinElute Gel Extraction Kit, Qiagen, 28604). Library preparation and quality control as well as amplicon sequencing (Illumina MiSeq V3 technology, 300 bp, paired-end reads) of the VH and VL+CDRH3 libraries were performed at LGC Genomics. Sequence data were pre-processed by LGC Genomics according to their standard procedure followed by in-depth analysis (custom made, proprietary) by Bionamic Data Consulting. In brief, VH and VL+CDRH3 sequences were combined based on CDRH3 sequence to obtain the full scFv gene. In cases where more than one CDRH3 variant was found in otherwise identical VH or VL+CDRH3 sequences, the CDRH3 frequencies in the two libraries were used to join the correct VH/VL pair. For each sequence, the number of reads in the various phage pools was normalized based on the total number of reads in each specific library before comparison. The number of reads in the different tracks is summarized in **Table 1**. Sequences with less than two reads were omitted from the analysis. A search in the NGS data was done for all clone sequences discovered previously (Ljungars et al., 2018) or in this study through Sanger sequencing and used for evaluation of the deep mining strategies.

**Table 1:** Number of reads in next-generation sequencing (NGS) raw data from the various tracks including the final number of reads used for analysis.

	VH raw data (no. of reads)	VL+CDRH3 raw data (no. of reads)	VH and VL complete sequence (no. of reads)
Input	1,811,814	3,558,517	967,981
Track B1	1,581,405	2,640,958	618,079
Track B2	1,745,869	2,465,931	802,039
Track PD	1,713,997	2,780,096	755,186
Track C	1,853,744	3,117,052	542,196

### Synthesis of Clones and Production of Human IgG

Based on the frequency in the various phage pools, 50 sequences, previously not found, were selected for synthesis. Selected DNA genes were synthesized (Twist Bioscience) and ligated into a proprietary in-house developed vector containing genes encoding the heavy and light chain constant regions of a human IgG1 antibody and produced and purified as described previously (Ljungars et al., 2018).

### Binding Analysis of Human IgG1 Antibodies

Produced purified human IgG1 antibodies were labeled with biotin using a Zenon kit (Thermo Fisher Scientific, Z25452) and a left to bind, 10 µg/ml, 25 µl/well, CLL cells from four patients and PBMC from two healthy donors (100,000 cells/well) for 1 h at +4°C. Following washing, binding was detected with Streptavidin-AF647 (Thermo Fisher Scientific, S32357). Cells were stained with SYTOX green (Thermo Fisher Scientific, S7020) to allow gating of live cells, and PBMC was, in addition, stained with anti-CD19-PE (BD Biosciences, 555413) for gating of CD19− cells. Finally, cell binding was analyzed in flow cytometry (FACS Verse, BD Biosciences).

## Results

### Discovery of Low-Abundant Antibodies Through Fishing Using Recombinant Proteins

**Figure 3:**
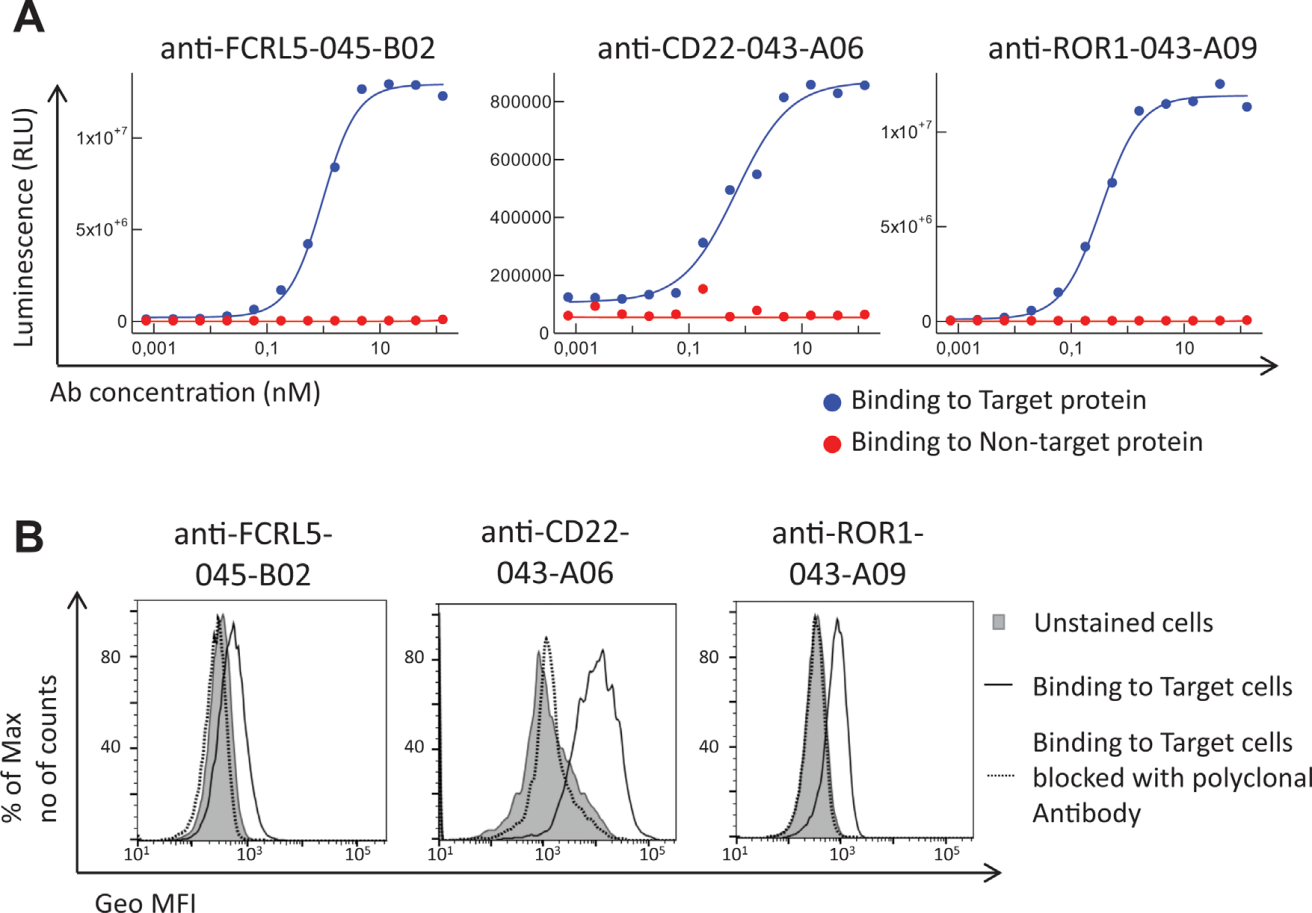
Binding analysis of antibodies generated through fishing with recombinant CD22, ROR1, and FCRL5 proteins. **(A)** Dose–response ELISA of one representative antibody (human IgG1) per protein. Each antibody was analyzed for binding to the specific protein used for fishing (target protein) and a non-related protein carrying the same tag (non-target protein). Binding was detected using an HRP-labeled anti-human antibody and a luminescent substrate. **(B)** Flow cytometry analysis of one representative antibody (human IgG1 or scFv) per target showing the binding to target cells, that is, chronic lymphocytic leukemia (CLL) cells (anti-FCRL5) or Raji cells (anti-CD22 and anti-ROR1), with or without a prior blocking step with a commercial polyclonal antibody of the same specificity as the tested antibody. Binding was detected using an APC-labeled anti-human antibody (anti-CD22 and anti-ROR1) or an AF647-labeled anti-tag antibody (anti-FCRL5).

Antibodies targeting additional cell surface receptors, not previously discovered through direct screening (traditional immunochemical screening of a few thousand randomly selected clones), are likely available in the anti-CLL phage pool. To facilitate discovery of such rare clones, various deep mining strategies, all depicted in **Figure 2**, were applied. As a first strategy, recombinant proteins corresponding to five previously undiscovered receptors with a high gene expression ratio between target (CLL) and non-target cells (B-cell-depleted PBMC) and confirmed cell surface expression on CLL cells were selected for fishing (track F). After screening of 192 clones per protein in ELISA, unique clones identified through Sanger sequencing were purified, and binding was confirmed in a dose–response ELISA. High-affinity antibodies (**Table 2**) targeting three of the receptors CD22 (*n* = 1), ROR1 (*n* = 5), and FCRL5 (*n* = 14) were identified (**Figure 3A**). A few clones, at least one per receptor, were analyzed for binding to CLL cells. All analyzed antibodies bind to CLL cells (data not shown), and their specificity was confirmed by blocking of cell binding with a polyclonal antibody targeting the same antigen (**Figure 3B**).

**Table 2:** EC_50_ values in dose–response ELISA for clones identified through fishing.

Receptor	Clone	EC_50_ (nM)
FCRL5	045-D05	8
FCRL5	044-B05	40
FCRL5	044-H01	3
FCRL5	045-E03	30
FCRL5	044-B02	0.3
FCRL5	044-A02	0.1
FCRL5	044-D02	2
FCRL5	044-H04	0.9
FCRL5	045-B06	2
FCRL5	044-E03	0.3
FCRL5	045-C02	0.2
FCRL5	045-B02	0.7
FCRL5	044-C04	0.2
FCRL5	045-D01	0.2
ROR1	043-A10	0.2
ROR1	045-A10	0.1
ROR1	045-E10	20
ROR1	043-A09	1
ROR1	043-E09	0.4
CD22	043-A06	0.7

The number of CD22, ROR1, and FCRL5 receptors, to which binders were present at low frequency in the pool, was determined on target and non-target cells. CD22 and ROR1 are expressed at approximately 6,000 and FCRL5 at 1,000 receptors per CLL cell, respectively (**Table 3**), numbers substantially lower than receptors for which scFv was found through direct screening (Ljungars et al., 2018). Importantly, fishing with recombinant proteins demonstrated that the anti-CLL phage pool contained antibodies binding low-abundant cell surface receptors, and such antibodies require powerful deep mining strategies to be discovered.

**Table 3:** Protein and gene expression levels in chronic lymphocytic leukemia (CLL) cells (target cells) and CD19− PBMC from healthy donors (non-target cells) for receptors successfully used in fishing.

Receptor	Protein expression		mRNA expression
	No. of receptors/target cell (*n* = 9)	No. of receptors/non-target cell (*n* = 6)	Target/non-target ratio
FCRL5	1,000	400	45
CD22	6,000	300	26
ROR1	6,000	2,000	35

### The Effect of Antibody Blocking and Protein Depletion With Respect to Specificity in Generated Antibody Pools

**Figure 4:**
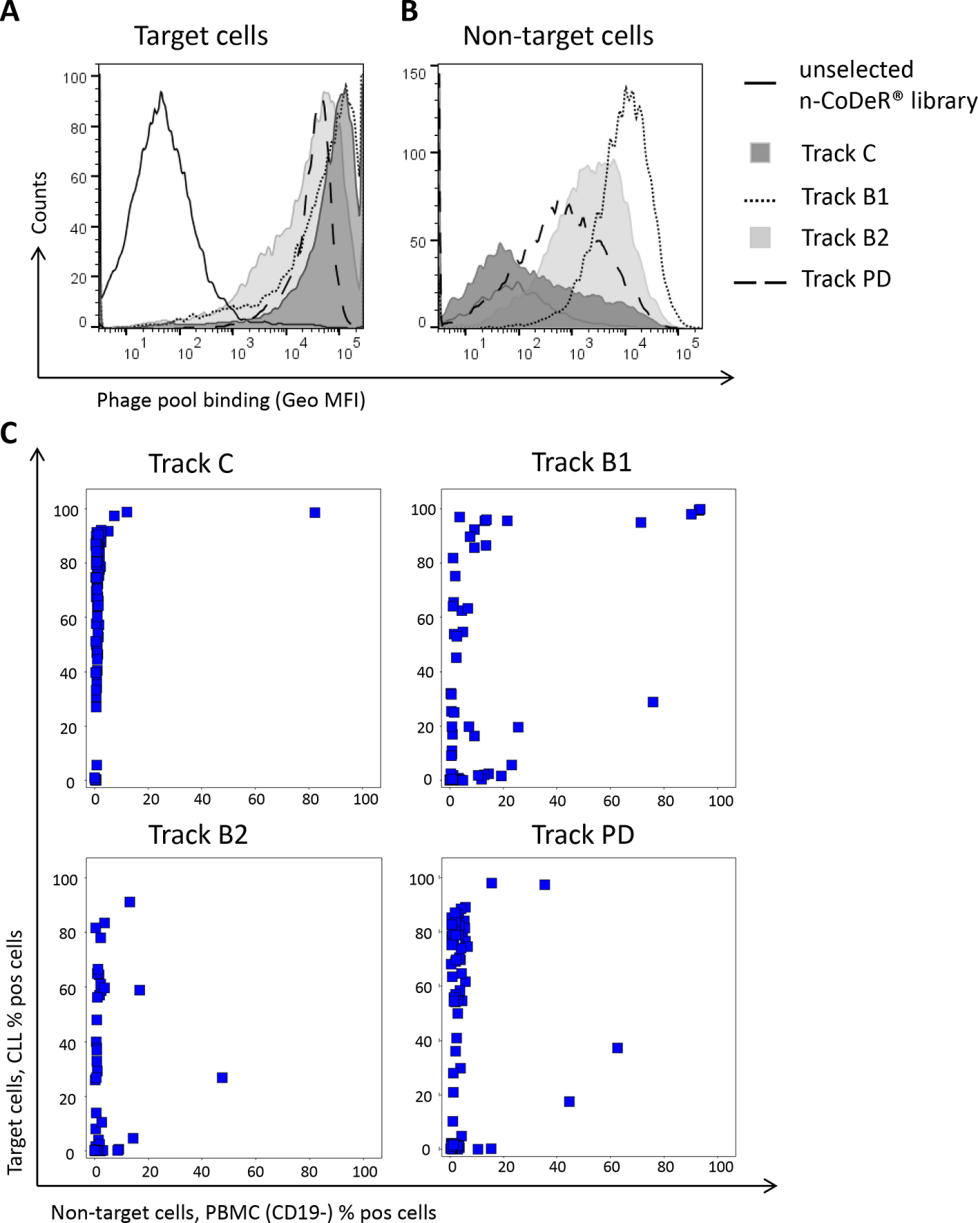
Phage pool binding in flow cytometry to **(A)** CLL cells (target) and **(B)** B-cell-depleted PBMC (non-target) for amplified phage pools from antibody blocking (tracks B1 and B2) and protein depletion (track PD) including the naïve library (n-CoDeR^®^) and the control panning (track C) for comparison. Binding was detected using an AF647-labeled anti-M13 antibody. **(C)** Screening in flow cytometry of 96 individual soluble scFv from tracks C, B1, B2, and PD showing the binding to target and non-target cells. Binding was detected using an AF647-labeled anti-tag antibody.

To reduce the frequency of dominating antibodies in the anti-CLL phage pool and thereby facilitate discovery of new clones, antibody blocking and protein depletion were performed as depicted in **Figure 2**. The specificity of the output phage pools, including track C, was analyzed for binding to target (CLL) and non-target cells (B-cell-depleted PBMC) by flow cytometry. Phage pools from both antibody blocking and protein depletion bound strongly to the target cells; pools from tracks B2 and PD were somewhat weaker than the pool from track B1, which was comparable with the control (**Figure 4A**). Binding to non-target cells was much increased for phage stocks resulting from selection that included steps of antibody blocking or protein depletion compared with the control (**Figure 4B**). As expected, phage pools from tracks B2 and PD, including a non-target cell depletion step, showed reduced non-target cell binding compared to track B1, where no such depletion had been performed.

**Figure 5:**
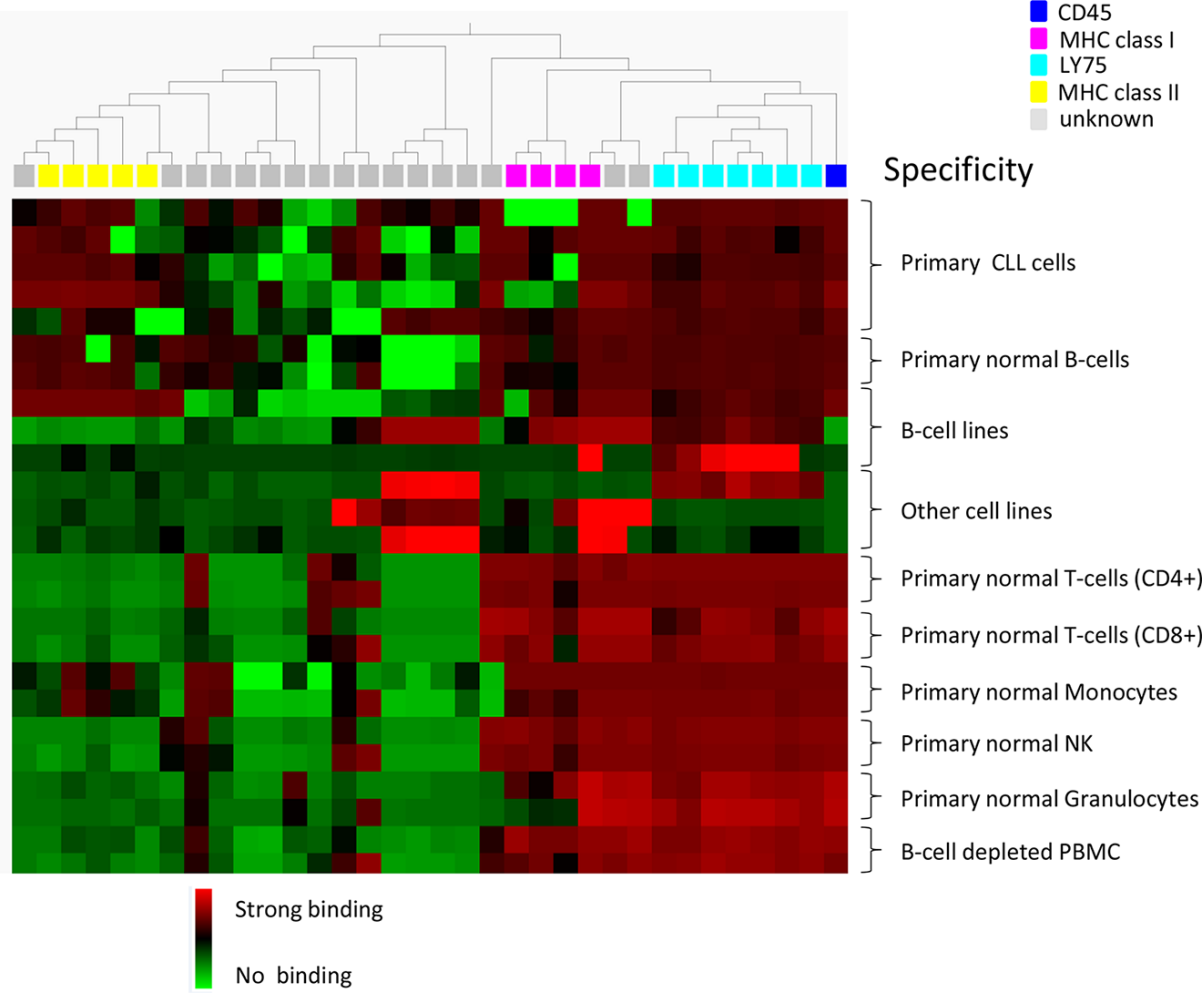
Heat map showing the binding pattern of 34 scFv in flow cytometry to CLL cells from five patients, B-cells (CD19+), CD4+ and CD8+ T-cells, monocytes (CD14+), NK cells (CD56+), granulocytes, and B-cell-depleted PBMC from two healthy donors, B-cell lines (Raji, RPMI, and WEHI) and other cell lines (Lovo, MCF-7, and DU145). Binding was detected using an AF647-labeled anti-tag antibody. Based on binding pattern, a hierarchal clustering of antibodies was made using Qlucore™ Omics Explorer. The binding intensity is color coded based on relative signal intensities within each cell type, where red represents the strongest binding, green no binding, and black in between. Antibody specificity is shown by color coding on top of the heat map.

Individual clones, in scFv format, from the cell pannings (96 clones from each track) were also analyzed for binding to target and non-target cells using flow cytometry. As expected, the number of scFv binding with a high signal to CLL cells decreased with antibody blocking and protein depletion compared with the control (**Figure 4C**). The number of scFv binding to non-target cells was slightly higher in track B1 compared with tracks B2 and PD, in agreement with the pool analysis. The phenomenon, with some clones binding also to non-target cells after antibody blocking, was further confirmed in a separate deep mining experiment performed according to track B1, but using CLL cells from other patients. Individual scFv was isolated, and unique CLL-binding clones were analyzed for binding to CLL cells from several patients, cell populations in PBMC, and some cell lines. The cell binding analysis showed that removal of the most abundant clones through antibody blocking resulted in some scFv that bound to both the CLL target cells and the non-target cells (**Figure 5**). Target deconvolution for a part of these antibodies discovered binders specific for LY75, CD45, MHC class I, and MHC class II, antigens that are commonly present on many cell types. It is thus useful to include a depletion step with non-target cells in the deep mining strategies to counteract enrichment of clones binding both target and non-target cells.

**Figure 6:**
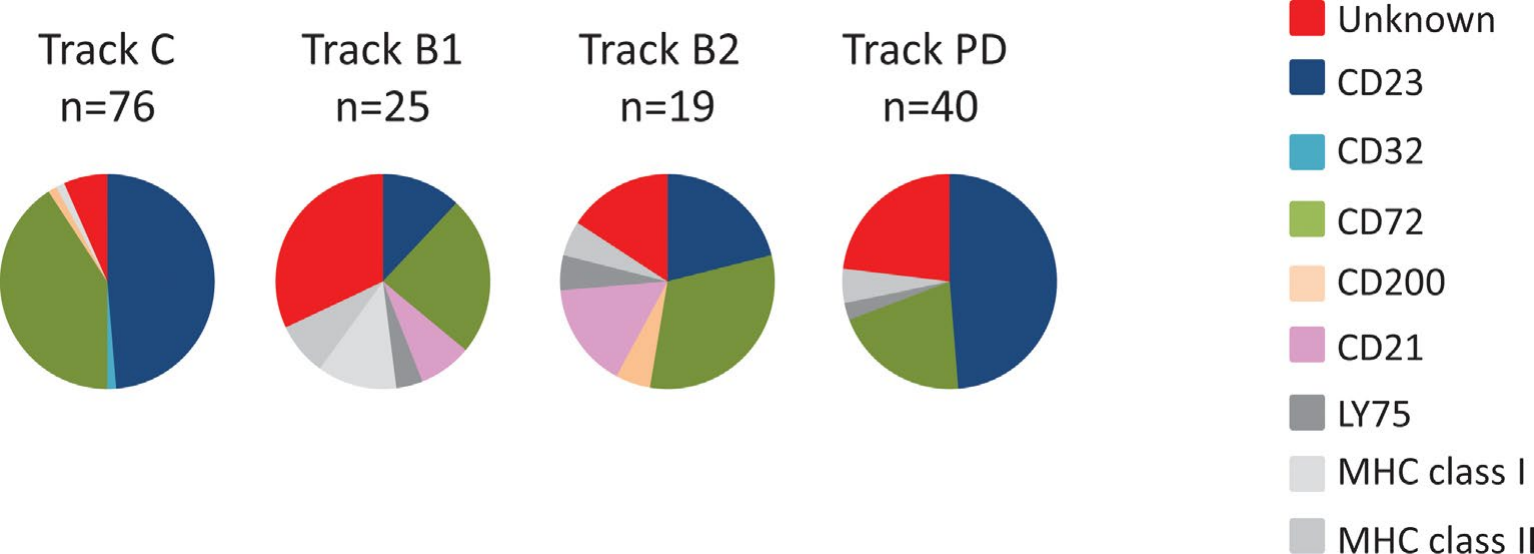
Target specificity distribution, as determined by ELISA, for unique clones from track C, track B1, track B2, and track PD. Binding to coated proteins was detected with AP-labeled anti-tag antibody and a luminescent substrate.

To evaluate the specificity of clones from antibody blockings and protein depletion, genes encoding scFv binding to more than 20% of the CLL target cells were sequenced. All unique scFv was then analyzed for binding to previously discovered targets, including the ones expressed on both target and non-target cells, using recombinant proteins in ELISA. This showed that the antibodies discovered through tracks B1, B2, and PD have a different target specificity distribution than has track C (**Figure 6**). Whereas track C was dominated by antibodies targeting CD23 and CD72, antibody blocking reduced the fraction of antibodies binding to these two targets significantly. In track B1, as seen above, the fraction of clones binding to cell surface receptors expressed on cell populations other than the target cells increased, here illustrated by antibodies binding to LY75, MHC class II, and MHC class I. This number was reduced in track B2, confirming the effect of the non-target cell depletion step. The protein depletion step mainly reduced the fraction of CD72 binding antibodies, indicating that the efficacy of protein depletion can vary depending on the protein and the methodology used. Importantly, both antibody blocking and protein depletion increased the fraction of antibodies binding to, for this phage pool, unknown, previously not identified, cell surface receptors.

### NGS Analysis of Antibody Enrichment in Antibody Blockings and Protein Depletion Strategies

**Figure 7:**
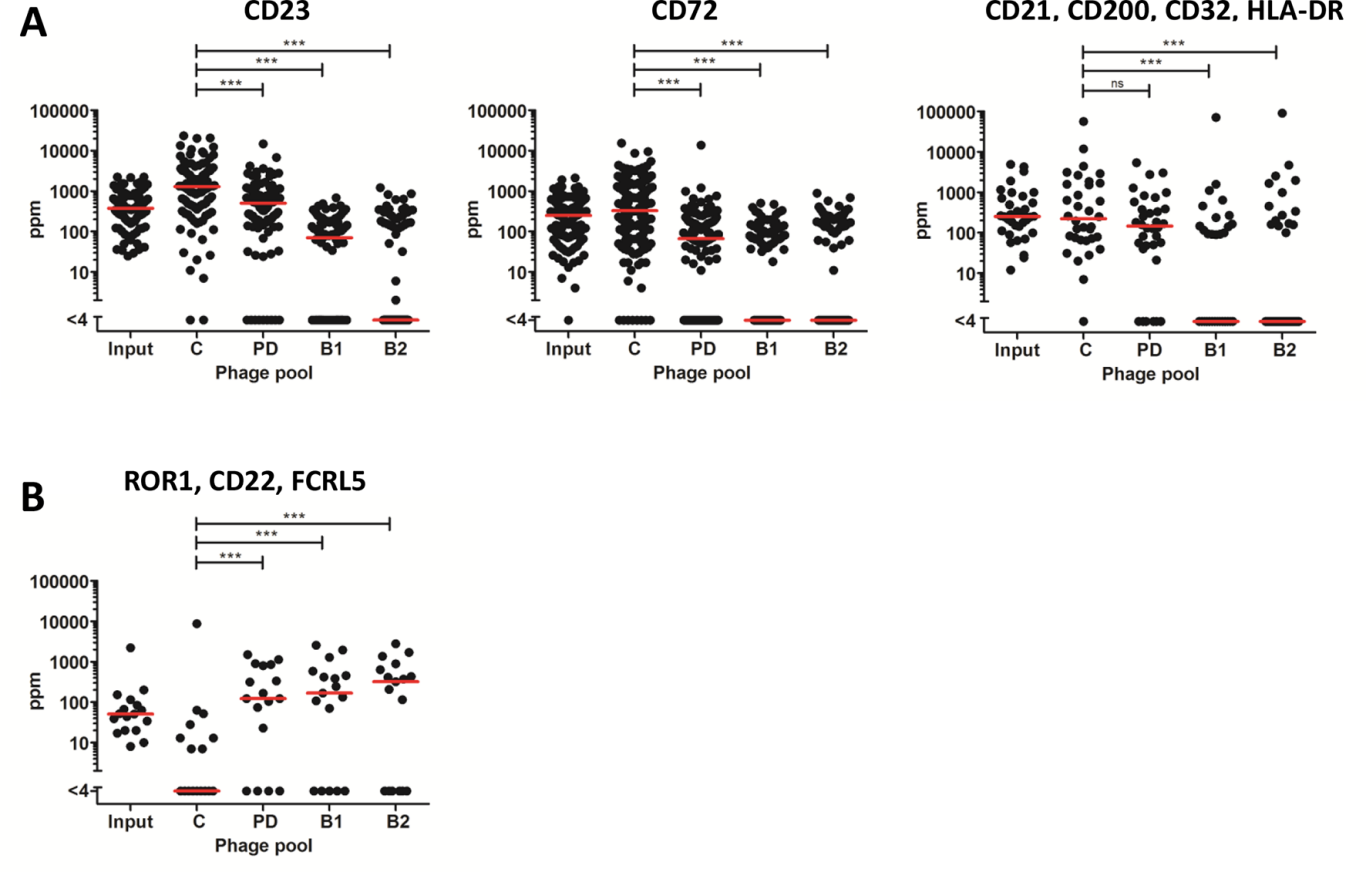
Frequency, based on NGS analysis, of discovered clones in the different strategies including the input phage pool. Median frequency is shown with red bars. **(A)** Antibodies, previously discovered through direct screening with corresponding specificities, CD23 (*n* = 107), CD72 (*n* = 132), and CD200, CD21, CD32, or HLA-DR (*n* = 34). **(B)** Antibodies, discovered through fishing, binding FCRL5, ROR1, or CD22 (*n* = 17). For statistical analysis, Friedman’s test with Dunn’s multiple comparison was done using GraphPad Prism.

NGS offers an opportunity to assess clonal selection at great depth. The input phage pool and the output phage pools from tracks B1, B2, PD, and C were analyzed by NGS to evaluate how the deep mining strategies affected the enrichment of clones. Importantly, the frequency of antibodies targeting the six cell surface receptors previously discovered through direct screening (CD21, CD23, CD32, CD72, CD200, and HLA-DR) was reduced in the antibody blocking tracks (**Figure 7A**). A similar effect was seen in track PD for antibodies binding to receptors included in the protein depletion. As a contrast, antibodies otherwise only discovered through fishing (track F), binding CD22, FCRL5, or ROR1, were very rare in the control panning but increased between 100 and 300 times by protein depletion or antibody blocking strategies (**Figure 7B**).

### NGS Analysis as a Deep Mining Strategy

**Figure 8:**
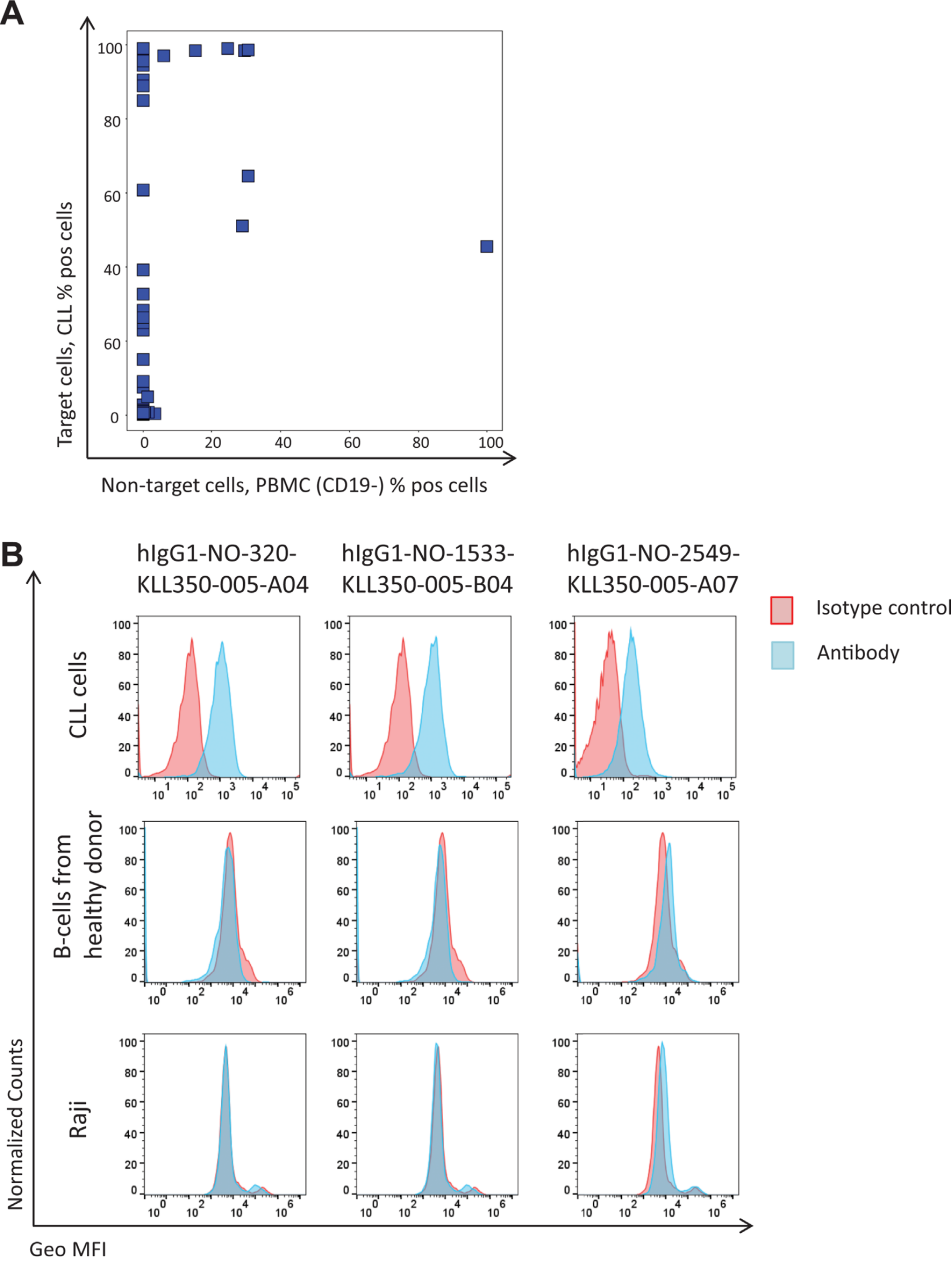
Flow cytometry binding analysis of antibodies corresponding to antibody genes identified through NGS. Antibody genes were synthesized and used for production of human IgG1 antibodies. Purified antibodies were labeled with biotin using a zenon kit followed by Streptavidin-AF647 detection. **(A)** Binding to target versus non-target cells for produced antibodies (*n* = 43). **(B)** Binding pattern, for three of the antibodies to CLL cells, B-cells (CD19+) from a healthy donor, and a B-cell line (Raji).

The NGS analysis demonstrated that the protein depletion and antibody blocking strategies altered the phage pool, and as a result, most rare clones increased in frequency, as expected. Based on this finding, 50 sequences, previously not found, with an increased frequency in the phage pools from protein depletion or antibody blockings and unaffected by a non-target cell depletion step (similar frequency in tracks B1 and B2) were selected for DNA synthesis and protein expression (**Supplementary Table 1**). Most of the antibodies, 43, were successfully produced as human IgG1s. All IgGs were analyzed for binding to previously discovered targets in ELISA, where only two of the clones showed binding to CD21 and CD200; data not shown. As a pool of CLL cells from several patients was used to generate the input phage pool, with another pool of cells used for pannings in this study, it is possible that rare antibodies bind an antigen that is only present on a subset of the patient cells. The binding of antibodies was therefore evaluated by flow cytometry to CLL cells from four patients, and 32 showed binding to CLL cells from at least one of these. The majority of the antibodies showed no binding to the non-target cells (B-cell-depleted PBMC) (**Figure 8A**). A few CLL-binding clones showed no binding to B-cells from healthy donors or a B-cell line (**Figure 8B**) and also no binding to other cell types in PBMC from healthy donors (T-cells, NK cells, monocytes and granulocytes; data not shown), indicating binding to a CLL-specific target. This demonstrates the capability of the NGS analysis to discover clones of low abundance (around 0.005% in track C) that are highly specific for the target cells.

### NGS Analysis of the Antibody Blocking Strategy

**Figure 9:**
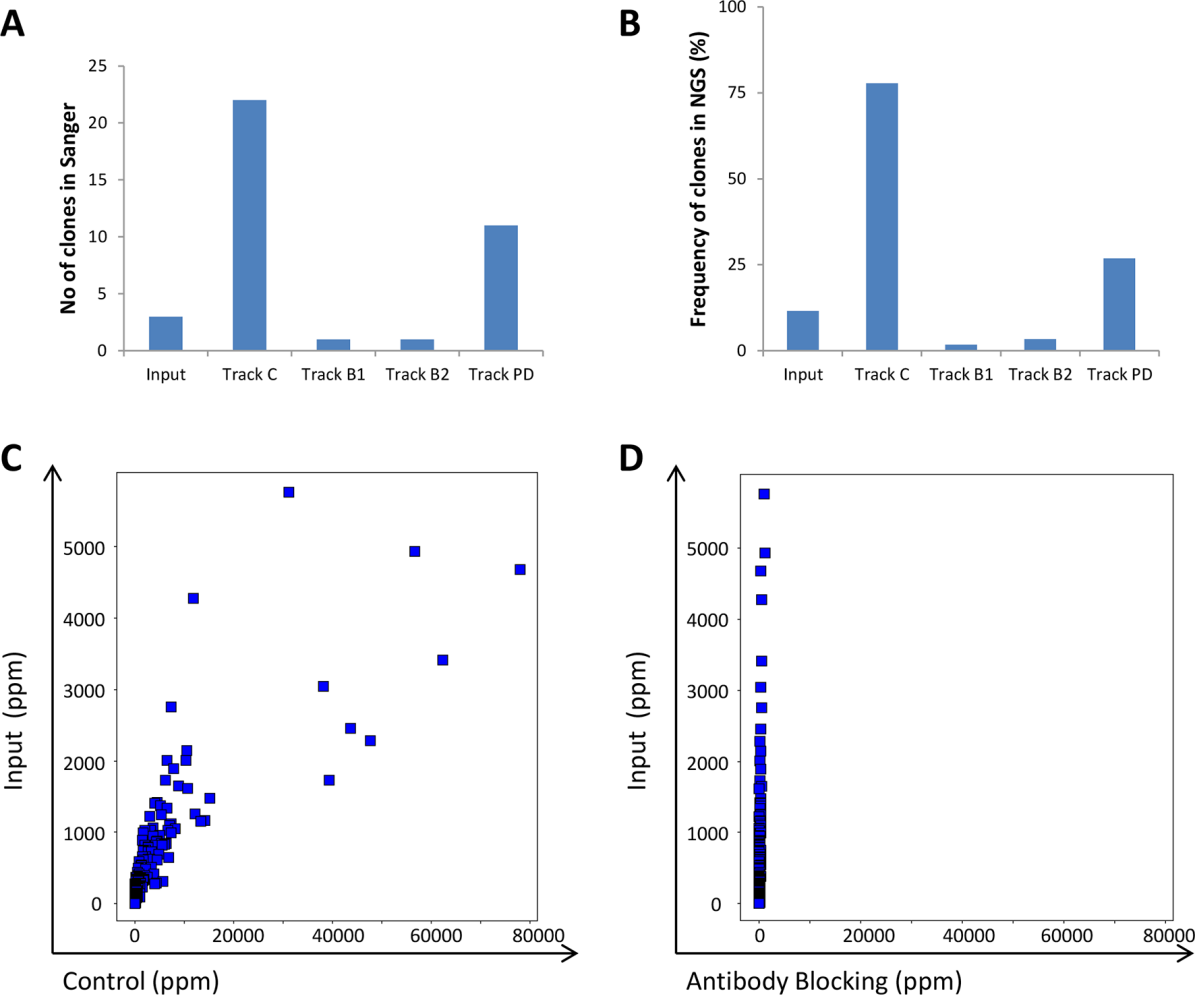
Analysis of the 241 clones included in the IgG pool used for antibody blocking. **(A)** Number of clones that were rediscovered in Sanger sequencing of CLL binding clones found through screening of 96 clones/track. **(B)** Total fraction of the 241 clone sequences based on NGS analysis of the phage pools. The frequency for all 241 individual antibodies in **(C)** input versus control or **(D)** antibody blocking phage pools, based on NGS analysis.

To evaluate the efficacy of the antibody blocking strategies, both the input and the output from the various strategies were analyzed using both traditional Sanger sequencing and NGS. We hypothesized that blocking with IgG during the panning process should reduce the fraction of phages displaying scFv corresponding to these IgGs in the output pool. This was indeed observed in the limited number of individual clones analyzed by Sanger sequencing after screening of 96 clones/track (**Figure 9A**) and was further confirmed by the NGS analysis (**Figure 9B**). The control strategy showed that without antibody blocking, the 241 clones (Ljungars et al., 2018) increased in frequency compared with the input material (**Figure 9C**) and together constituted almost 80% of the output of track C (**Figure 9B**); but with antibody blocking, all 241 clones showed a reduced frequency (**Figure 9D**).

**Figure 10:**
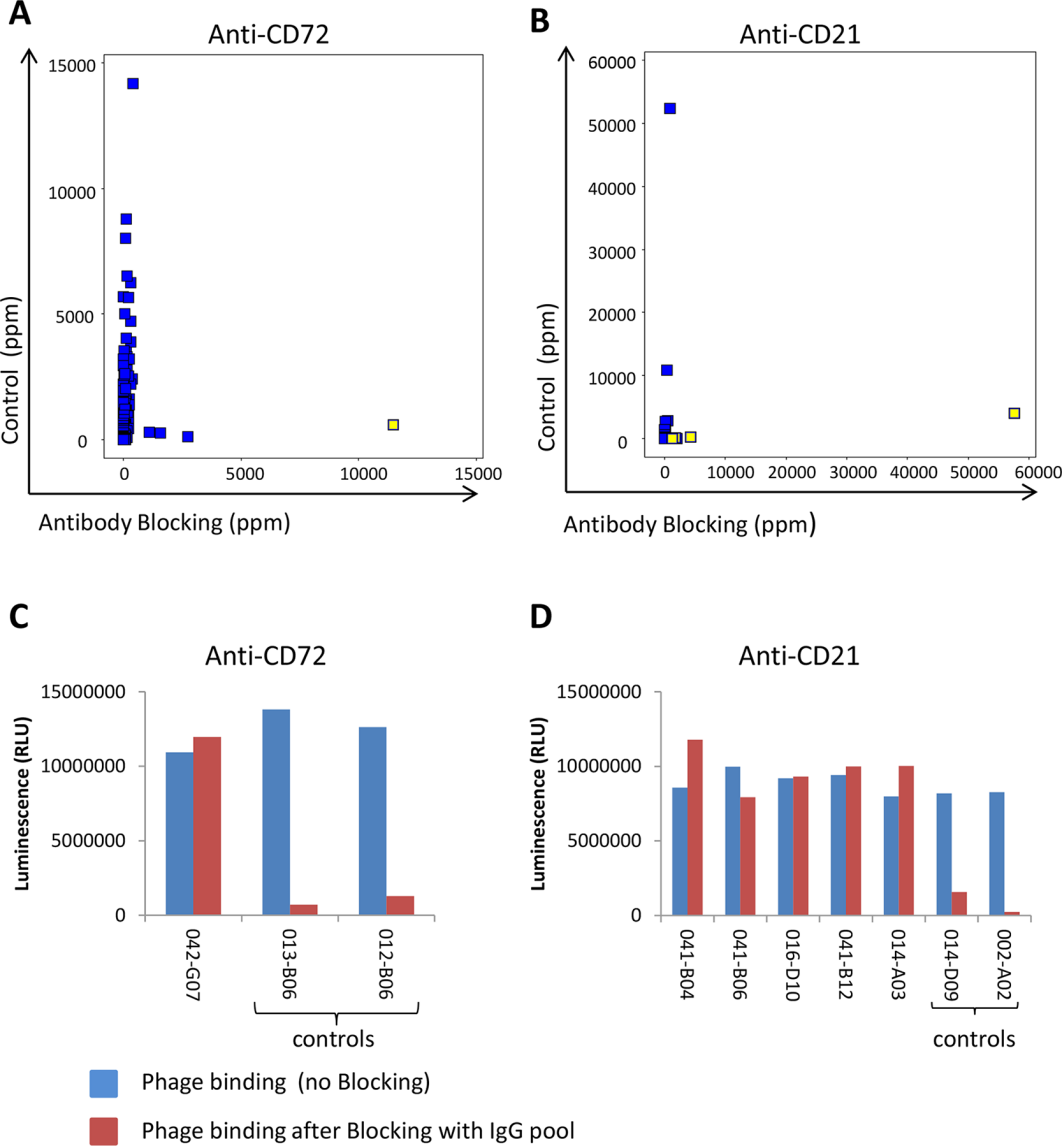
NGS analysis of identified antibodies binding to **(A)** CD72 (*n* = 301) or **(B)** CD21 (*n* = 27) showing the frequency in control (track C) versus antibody blocking (track B1). Yellow-marked clones were analyzed for binding, as scFv displayed on phage, to coated **(C)** CD72 or **(D)** CD21 protein in ELISA with or without prior blocking with the pool of IgGs used for blocking in tracks B1 and B2. As control, two additional clones per target, corresponding to sequences showing a decreased frequency in tracks B1 and B2 in the NGS analysis, were included. Binding was detected using an HRP-labeled anti-M13 antibody and a luminescent substrate.

In addition to preventing the same clones from being rediscovered and to allow discovery of antibodies to new targets, antibody blocking also enabled discovery of low-abundant antibodies binding to new epitopes of previously identified targets. The frequency for clone sequences corresponding to binders specific for the initially six discovered cell surface receptors was compared between tracks C and B. The vast majority of clones showed a reduced frequency by antibody blocking. However, a few clones binding to CD72 and CD21 instead increased in frequency by antibody blocking (**Figures 10A, B**). Phages displaying scFv corresponding to these clones were analyzed for binding to CD72 and CD21 in ELISA with or without pre-incubation with the IgG pool used for blocking during the panning. The result showed that these clones bind regardless of whether the IgG pool was added or not, which demonstrates binding to other epitopes than the antibodies included in the IgG pool (**Figures 10C, D**). As control, a few phages displaying scFv corresponding to clones with reduced frequency in the antibody blocking strategies compared with the control were included. These control clones showed no binding after pre-incubation with the IgG pool, as expected. Altogether, these results demonstrate that an antibody blocking strategy facilitate discovery of antibodies that are not easily found by conventional selection strategies.

### Comparison between the Deep Mining Strategies With Respect to Discovery of Low-Abundant Clones

**Figure 11:**
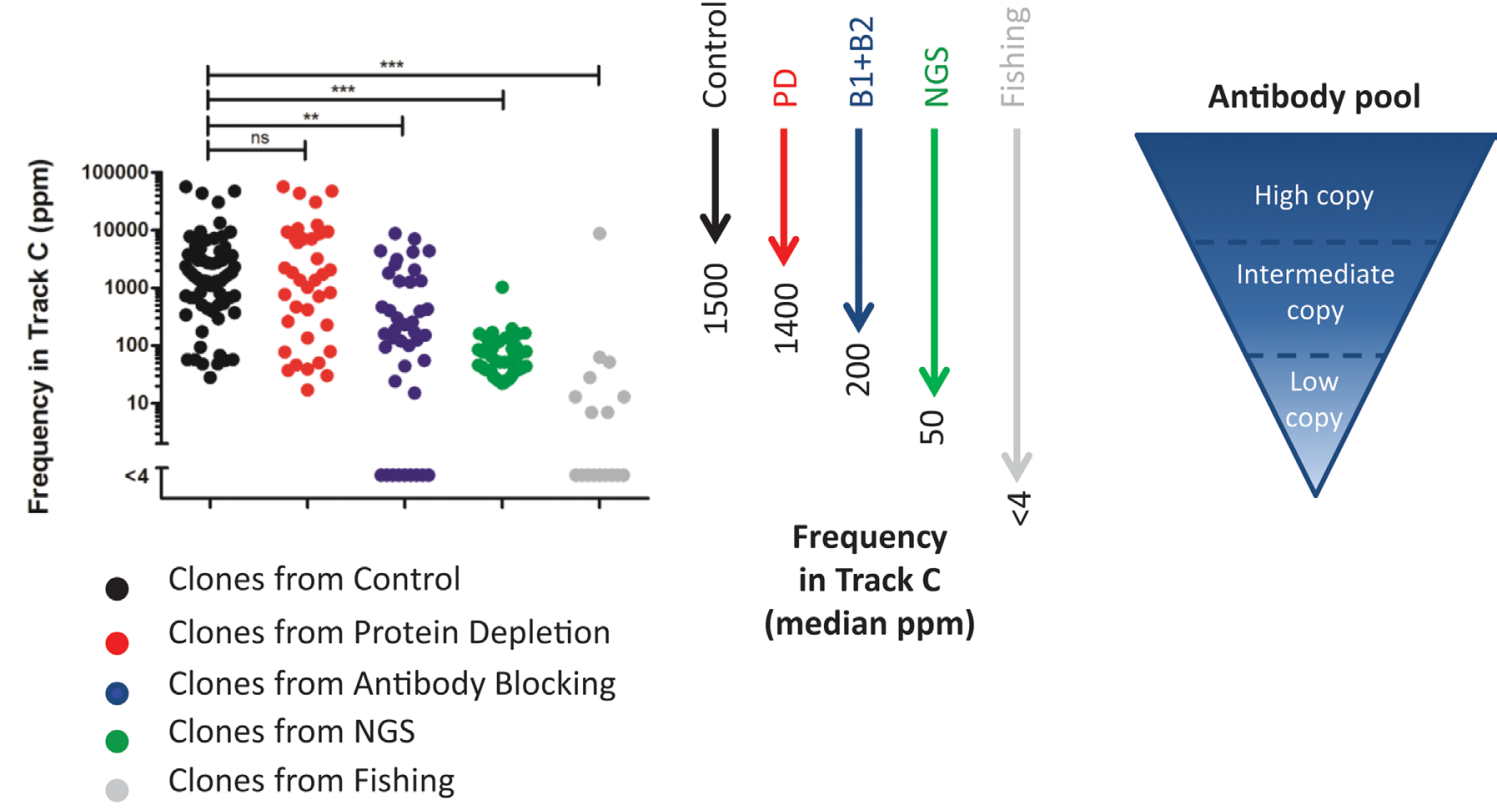
Frequency in track C for identified unique clones. The clones were found in track C (*n* = 70), track PD (*n* = 37), track B1 + B2 (*n* = 41), NGS (*n* = 50), or track F (*n* = 17). Unique clones not discovered in the NGS data from any phage pool were omitted from the analysis. For statistical analysis, Kruskal–Wallis test with Dunn’s multiple comparison was done using GraphPad Prism.

To compare the deep mining strategies and find out how deep they can reach into a complex antibody pool, the NGS results of the output pools were examined. Sequences representing unique clones, found through (1) screening of 96 clones/track, (2) NGS analysis followed by synthesis and production, or (3) fishing with recombinant proteins were identified in the NGS data set from track C (**Figure 11**).

In this comparison, clones found from the control pool, representing direct screening, have, as expected, the highest median frequency, 0.15%, in track C. A protein depletion or an antibody blocking step enables discovery of clones that have slightly lower or about 10 times lower frequency in track C, respectively. The clones selected for synthesis and production, based on NGS, have a median frequency of 0.005% in track C. These clones were however selected based on their abundance in various strategies, and also, clones of lower frequency can be identified with NGS. Antibody sequences discovered through fishing show a very low median frequency, less than 0.0004%, in track C. Although this fraction increased in tracks B1, B2, and PD (**Figure 7B**), fishing with recombinant proteins was needed to easily discover these clones.

The various strategies thus all reached different levels of the phage pool. By using a combination of direct screening and deep mining strategies, the full depth of an antibody pool generated through cell panning can be explored. This is important to increase target space and to find antibodies with the potential to be developed into novel, unique therapeutics that are not easily discovered by conventional methods.

## Discussion

For discovery of therapeutic antibodies through phage display technology, antibodies against a target, such as a protein or a cell, are enriched through consecutive selection rounds. This often results in a large phage pool consisting of antibody sequences of both high and low frequency (Yang et al., 2017). Direct screening, where only a sub-fraction of the antibody diversity is subjected to testing, mainly identifies the most abundant antibodies and, when whole cells are used as target, subsequently their corresponding specificities. As the antibody frequency is not related to functionality, there is a need to facilitate discovery also of low-abundant antibodies and thus find additional antibodies and, when applicable, additional specificities.

In this study, we evaluated four deep mining strategies to facilitate discovery of low abundant clones, using a phage pool previously generated by panning on a complex target, whole cells (Ljungars et al., 2018). From this pool, a diversity of binders with a handful of different specificities had been found through a direct screening approach (Ljungars et al., 2018). Yet the present study demonstrated that there were additional antibodies of different specificities to be found, at low frequency, as such antibodies could be enriched following selection on recombinant antigen. Additional deep mining strategies were used to identify rare clones that bind, for this phage pool, previously undiscovered targets and epitopes. These strategies can be used to unlock the full potential of phage pools generated through panning on cells.

To reduce the frequency of already discovered antibodies and specificities, we applied an antibody blocking strategy where the previously generated phage pool underwent panning on whole cells in the presence of 241 previously discovered antibodies specific for six different receptors. Antibodies against new epitopes on the target cells were successfully enriched and subsequently discovered. This is in agreement with previous studies where antibodies against new epitopes on a single target protein (Ditzel et al., 1995; Ditzel, 2002; Tsui et al., 2002), a cell lysate (Sanna et al., 1995), or whole cells (Even-Desrumeaux et al., 2014) have been found through a blocking strategy. As antibodies targeting a single cell surface receptor can have completely different functionalities and mechanisms of action depending on epitope, as seen for the various antibodies specific for CD20 commonly used for treatment of CLL (Beers et al., 2010), these findings illustrate an approach to discover antibodies with rare epitope specificity also on complex targets like intact cells. Previous studies have demonstrated the efficacy of blocking strategies for selection on much less complex antigens through the lack of rediscovery of the blocking clones (Ditzel et al., 1995; Ditzel, 2002; Tsui et al., 2002). We showed by NGS that the frequency of sequences representing blocking antibodies was strongly reduced in phage pools isolated through an antibody blocking strategy. In addition to being very effective, this strategy has the advantage of enabling removal of functionally not interesting antibodies, also of unknown specificity, a particularly useful approach in phenotypic discovery strategies, where target deconvolution is laborious and costly (Schirle et al., 2012; Lee and Bogyo, 2013). Altogether, antibody blocking has the potential to enable discovery of antibodies targeting epitopes rarely detected by antibodies, either on antigens previously discovered through direct screening or on entirely new antigens.

One of the advantages of phage display technology, compared with animal immunization, is that it allows a variety of depletion and competition steps to be performed to guide the selection, for instance, to a particular domain of an antigen or to discriminate between highly homologous antigens, as splice variants or those carrying a particular post-translational modification. In this study, we successfully used a protein depletion step as a deep mining strategy to reduce the fraction of antibodies binding previously discovered targets. Importantly, antibodies against targets that are known to be of low therapeutic interest can be removed by this method (Williams et al., 2016), thereby focusing the output onto other receptors displayed by the cell population used for selection. One should however be careful with which proteins to include in a depletion step of a phenotypic discovery as one advantage of this procedure is to find new mechanisms of action for previously known targets (Fransson et al., 2006; Veitonmaki et al., 2013; Roghanian et al., 2015; Ljungars et al., 2018). Overall, protein depletion can be used to reduce the frequency of antibodies of certain specificity without prior generation of antibodies, as a complement to antibody blocking.

NGS has previously been used for discovery of antibodies from phage display libraries (Ravn et al., 2010; Zhang et al., 2011; Ravn et al., 2013; Lopez et al., 2017; Yang et al., 2017; Stutz et al., 2018; Barreto et al., 2019). These studies have shown that compared with traditional screening, NGS enables discovery of more clones (Ravn et al., 2010; Ravn et al., 2013; Yang et al., 2017; Stutz et al., 2018), clones with higher affinity (Barreto et al., 2019), or clones binding functionally more interesting epitopes (Lopez et al., 2017; Stutz et al., 2018; Barreto et al., 2019). In many of these studies, the most enriched antibody sequences, against one or several proteins, although sometimes in a complex context (Zhang et al., 2011), have been recovered and used for downstream analysis. In this study, we expanded the use of NGS for phage display antibody discovery and analyzed complex phage pools generated through cell pannings to evaluate not primarily the most abundant clones that had largely already been discovered by direct screening but instead the low-abundant clones that had been enriched through deep mining strategies. The majority of these antibodies bound to the target cells with no binding to the non-target cells demonstrating selectivity also of low-abundant clones. NGS is thus very powerful as a deep mining strategy and allowed us to identify clones of very low frequencies. A limitation of pure NGS-based screening is currently the cost of gene synthesis and lack of direct evidence of binding specificity of identified clones. However, a reduced cost of gene synthesis in combination with the capability to synthesize thousands of genes (Kuhn et al., 2017) will likely make this approach even more useful for phage display antibody discovery in the future.

One challenge in the discovery of antibodies against low expressed cell surface receptors is the sensitivity of the assay used for detection of antibody binding. Antibodies to really low expressed receptors, such as the ones discovered through fishing in this study, would have been missed using a flow cytometry screening on primary cells alone, since the signal is close to background. The number of clones binding to targets with a low expression level on the cells increases in the deep mining strategies (**Figure 4C**) and is therefore most likely higher than the number of clones selected for sequencing in this study. Using a more sensitive assay (Earnshaw and Osbourn, 1999; Gusev et al., 2001), and/or more allowing criteria in the screening, probably would have resulted in identification of even more clones binding to new, currently unknown, targets.

In summary, a phage pool generated through a cell panning strategy is usually a selective, complex population of binders, many of which are beyond reach when using a discovery approach based on direct screening alone. We show that the combination of direct screening and deep mining enables discovery of clones of both high and low frequencies, which allows additional targets and epitopes to be found. Our findings have the potential to advance discovery of novel targets, pathways, and antibodies to facilitate development of new treatments for patients with various diseases.

## Data Availability

All datasets generated for this study are included in the manuscript/**Supplementary Files**.

## Author Contributions

AL designed the study, performed research, analyzed data, and wrote the manuscript. CS performed research and analyzed data as part of a defended Master’s thesis. AC analyzed data. EB performed research. U-CT and BF contributed to scientific discussions. MO and MM designed and supervised the study and edited the manuscript. All authors approved the final version of the manuscript.

## Funding

The project was in part supported by a grant from the Swedish Foundation for Strategic Research (MO).

## Conflict of Interest Statement

AL, CS, EB, U-CT, BF, and MM were employed at BioInvent International AB and AC was employed at Bionamic data consulting AB during their contribution to this paper.

The remaining author declares that the research was conducted in the absence of any commercial or financial relationship that could be constructed as a potential conflict of interest.
