# Supplementary figures and images for: Deep Mining of Complex Antibody Phage Pools Generated by Cell Panning Enables Discovery of Rare Antibodies Binding New Targets and Epitopes
Source: Front Pharmacol. 2019 Jul 30;10:847. doi: 10.3389/fphar.2019.00847 (PMC6683657; doi:10.3389/fphar.2019.00847)

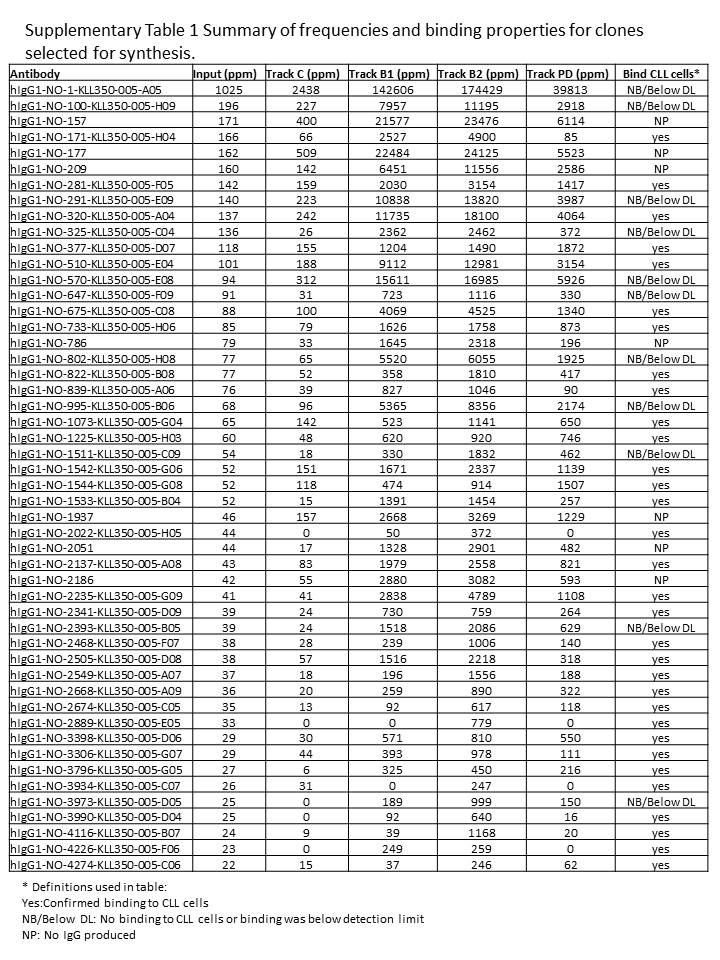

Supplement: Supplementary file 1 [file Image_1.jpeg]
